# Racial Differences in Chronic Stress/Allostatic Load Variation Due to Androgen Deprivation Therapy in Prostate Cancer

**DOI:** 10.1016/j.jaccao.2022.10.004

**Published:** 2022-10-31

**Authors:** Nickolas Stabellini, Jennifer Cullen, Justin X. Moore, Lifen Cao, Neeraj Agarwal, Nelson Hamerschlak, Kristin Waite, Alberto J. Montero, Jill S. Barnholtz-Sloan, Avirup Guha

Allostatic load (AL) is a score calculated through biomarkers involving the cardiovascular, metabolic, and inflammatory systems that reflects the cumulative effect of stress caused by adaptation to adverse psychosocial or physical situations.^1^ Non-Hispanic Blacks (NHBs) have a higher AL burden compared with non-Hispanic Whites (NHWs). A 1-unit increase in AL over a 9.9-year period can increase the risk of cardiovascular disease (CVD) by up to 130%.^2^ Androgen deprivation therapy (ADT), a cornerstone treatment for advanced prostate cancer (PC), is associated with increased CVD risk, with a significantly higher burden of CVD in NHBs compared with NHWs.^3^^,^^4^ We hypothesized that upon ADT initiation, AL increases more in NHBs compared with NHWs, leading to racial differences in CVD.

The study included men ≥18 years of age diagnosed with PC (2005-2022) at the University Hospitals (UH) Seidman Cancer Center, a large hybrid academic community practice. The study was approved by the UH of Cleveland Institutional Review Board. The UH Seidman Cancer Center database is available at UH Cleveland Medical Center with access restricted to researchers with Institutional Review Board approval.

AL (an ordinal measure from 0 to 11) was calculated before cancer diagnosis, and then monthly for the first year, assigning 1 point for the presence of each of the following cutoffs: systolic blood pressure ≥140 mm Hg, diastolic blood pressure ≥90 mm Hg, heart rate >100 beats/min, total cholesterol ≥240 mg/dL, high-density lipoprotein cholesterol ≤50 mg/dL, triglycerides ≥150 mg/dL, glycosylated hemoglobin ≥6.5%, body mass index ≥30 kg/m^2^, glucose ≥110 mg/dL, C-reactive protein >3 mg/L, and interleukin-6 >1.8 pg/mL.^1^ ADT use was defined by the prescription of 1 of the following: leuprolide, goserelin, triptorelin, degarelix, and regulolix.

The impact of different variables on monthly AL variation was assessed using general and race-specific generalized multivariable mixed-effects models. The models were adjusted for patient demographics, CVD risk factors, cancer characteristics, and cancer treatment information. Sensitivity analysis was performed with AL measured via the Chen et al^5^ methodology. The results were presented according to the least-squares mean with associated SEs.

A total of 7,168 PC patients were included, of which 1,570 were NHBs (Table 1). The median age was 68 (IQR: 62-75) years. Most patients had a Charlson score from 1 to 2 (59.3%), 14.4% patients had at least 1 CVD risk factor, and 12.7% had Gleason score ≥8. Surgery was performed in 25.8% of the cohort, 31.9% received radiotherapy, and 20.9% received ADT.

**Table 1:** Population Description, AL Components, its Changes Over Time, and the Impact of ADT

	All PC Patients (N = 7,168)	NHW Patients (n = 4,902)	NHB Patients (n = 1,570)	ADT Patients (n = 1,498)
Population description				
Age at diagnosis, y	68 (62-75)	69 (63-76)	66 (60-73)	—
Advanced TNM stage (IV)	443 (6.2)	131 (8.3)	278 (5.7)	—
High risk (Gleason ≥8)	902 (12.7)	212 (13.5)	610 (12.4)	—
Surgery	1,852 (25.8)	422 (26.9)	1,284 (26.2)	—
Radiotherapy	2,284 (31.9)	615 (39.2)	1,475 (30.1)	—
Chemotherapy	302 (4.2)	68 (4.3)	203 (4.1)	—
Multiple treatments	1,595 (22.3)	458 (29.2)	1,002 (20.4)	—
ADT	1,498 (20.9)	937 (19.1)	455 (29.0)	—
% of patients with AL components above the cutoff to assign 1 point to AL (pre-PC diagnosis → post-PC diagnosis at 1 year)				
Systolic blood pressure	44.1 → 57.2	45.6 → 59.3	47.4 → 60.6	44.9 → 64.0
Diastolic blood pressure	19.4 → 27.9	18.1 → 26.6	27.4 → 36.9	21.4 → 34.6
Heart rate	57.6 → 70.1	60.8 → 73.3	57.5 → 70.4	56.6 → 74.2
Total cholesterol	2.9 → 3.1	2.8 → 3.0	3.8 → 3.9	3.5 → 3.7
High-density lipoprotein	17.4 → 18.7	17.5 → 18.8	20.2 → 21.5	21.2 → 22.6
Triglycerides	9.7 → 10.4	10 → 10.9	9.6 → 10.4	11.3 → 12.3
Glycosylated hemoglobin	3.9 → 4.1	3.0 → 3.2	6.9 → 7.6	5.5 → 5.5
Body mass index	25.6 → 28.1	26.4 → 28.8	26.9 → 29.4	26.2 → 30.5
Glucose	48.3 → 59.6	48.8 → 60.5	53.6 → 64.1	48.7 → 62.2
C-reactive protein	4.4 → 5.9	4.0 → 5.4	5.9 → 7.7	4.7 → 6.2
Interleukin-6	0 → <1.0	0 → 0	0 → <1.0	0 → 0
AL variation over time				
AL pre-PC diagnosis	2 (0-4)	2 (0-4)	3 (0-4)	—
AL post-PC diagnosis (within 60 d)	3 (0-4)	3 (0-4)	3 (0-4)	—
AL post-PC diagnosis (at 1 y)	3 (1-4)	3 (1-4)	3 (1-5)	—
AL monthly variation in patients on ADT compared with those not on ADT—difference from least squares mean	0.14 ± 0.02 higher (<0.001)	0.16 ± 0.02 higher (<0.001)	0.10 ± 0.04 higher (0.025)	—

Values are median (IQR), n (%), or mean ± SE (*P*), unless otherwise indicated.

ADT = androgen deprivation therapy; AL = allostatic load; NHB = non-Hispanic Black; NHW = non-Hispanic White; PC = prostate cancer.

The median AL before the cancer diagnosis on the entire population was 2 (IQR: 0-4), and rose to 3 (IQR: 1-4) after the first year of diagnosis. NHBs had higher AL pre-PC diagnosis than NHWs (median AL pre-PC diagnosis: NHBs = 3 [IQR: 0-4]; NHWs = 2 [IQR: 0-4]; *P* = 0.001) (Table 1). Using generalized multivariable mixed-effects models, AL monthly variation was an estimated 0.14 ± 0.02 higher in the ADT relative to the no-ADT population (*P* < 0.001) (Table 1). NHBs on ADT had an average estimated AL monthly variation 0.10 ± 0.04 higher than those not on ADT (*P* = 0.025), while NHWs had an average estimated AL monthly variation 0.16 ± 0.02 higher than those not on ADT (*P* < 0.001) (Table 1). There were no racial differences between AL increase in those receiving ADT (0.08 ± 0.05; *P* = 0.080). Among components of AL, considering absolute values, diastolic blood pressure (43.0% increase), C-reactive protein (34.0% increase), and systolic blood pressure (29.0% increase) increased the most, and sensitivity analysis using the Chen et al^5^ methodology showed similar results.

Pre-PC diagnosis, chronic stress/AL is higher among NHBs vs NHWs. With ADT, AL increases similarly in both populations. PC patients treated with ADT experienced an increase in monthly AL between 0.10 and 0.16 compared with those untreated. No significant differences were observed according to self-identified race. Thus, precancer AL/level of chronic stress and non–ADT-related increase in AL may explain racial differences in ADT-related CVD.

Limitations of our study include the use of electronic medical record–based data, the single-institution setting, the inability to perform time-varying estimation on ADT, and a long time frame. Studies involving multiple institutions with prospective design, and including social determinants, are necessary to confirm and expand the findings.
